# How to define fish pathogen relatives from a 16S rRNA sequence library and Pearson correlation analysis between defined OTUs from the library: Supplementary data to the research article “Presence and habitats of bacterial fish pathogen relatives in a marine salmon post-smolt RAS”

**DOI:** 10.1016/j.dib.2022.108846

**Published:** 2022-12-31

**Authors:** K. Drønen, I. Roalkvam, K. Tungland, H. Dahle, H. Nilsen, A.B. Olsen, H. Wergeland

**Affiliations:** a Department of Biological Science, University of Bergen, Thormøhlensgt. 55, N-5020, Norway; b Independent Data Science Consultant, Equinor, PB 8500, N-4035, Norway; c Norwegian Veterinary Institute, Thormøhlensgt. 43C, N-5006, Norway

**Keywords:** Fish pathogens, 16S rRNA database (FPD), GenBank, Silva database

## Abstract

This paper provides supplementary data to the research paper ''Presence and habitats of bacterial fish pathogen relatives in a marine salmon post-smolt RAS" [1]. Here, environmental samples from a marine recirculating aquaculture system (RAS) were subjected to microbiome studies. This data article adds value to the research article by providing open access to data files that increased information retrieval from the 16S rRNA sequence library. A fasta file of full-length 16S rRNA sequences from fish pathogenic microbes was deposited in the Mendeley data repository, a collection named “Fish Pathogen Database”. Alignment of this database against the short sequences in the 16S rRNA library revealed the fish pathogen-relatives. Furthermore, a link to a CSV file containing Pearson correlation data was provided, an analysis based on the relative abundance information of all operational taxonomic units defined in the microbiome dataset. Included also, the methodological description of the Pearson correlation analysis, as well as a table where correlation data for the defined fish pathogen-relatives was retrieved from the large data file (Table 1).

**Specifications Table**

**Table:** 

Subject	Biological science
Specific subject area	Microbiology: Microbiome
Type of data	Given is the link to statistical Pearson correlation data of 535 operational taxonomic units (OTUs) defined in a microbiome study of a marine post-smolt RAS, i.e., taxonomic affiliation and relative abundances of samples from different sampling times and sampling sites. Thirteen of the OTUs obtained from the RAS were also defined as fish pathogen-relatives, as their sequences matched the 16S rRNA sequences of fish pathogenic bacteria in an alignment study. Provided is a link to the fasta file with the sequence collection from the fish pathogenic bacteria. Finally, a table of Pearson correlation data for the defined fish pathogen relatives is presented, information excerpted from the statistical dataset provided for all the 535 OTUs defined.
How data were acquired	Full-length16S rRNA gene sequences were retrieved from GenBank to the fish pathogen database directly or via the software SILVA 128. Silva has a quality threshold for the inclusion of new sequences to their database from GenBank, and thus recognized as a high sequence quality database. The GenBank and Silva platforms allowed sequences to be filtered by 16S rRNA gene identity and sequence length. Multiple sequences were included for each species, when available, as a strain may have multiple 16S rRNA gene operons. The selected sequences were downloaded in fasta file format and compiled into a single file as the fish pathogen database. The alignments between sequences in the fish pathogen database and the 16S rRNA gene sequence library from RAS (GenBank MN890148-MN891672) were performed in Linux. The Phyton/Pandas platform was used for the Pearson correlation analysis. In the latter case, OTU and relative abundance information was obtained from a CSV file previously deposited the Mendeley data depository with a url link published in the Data in Brief article:” Microbiome dataset from a marine recirculating aquaculture system (RAS) for salmon post-smolt production in Norway”[2] This data article also describes in detail the library preparation and sequencing platform used (Ion-Torrent).
Data format	The following files were in fasta format: *1524 sequences from the 16S rRNA sequence library *6750 sequences from 295 fish pathogenic bacteria. These were obtained from the GenBank/Silva database. Files created in excel format: *Overview table of the 6750 sequences given in the fish pathogen database. When depositing to Mendeley data, this information was provided in pdf format. *Taxonomic information for 535 OTUs from the 16S rRNA library. The text information was listed with a numerical code. The numerical code replaced the textual information in the CSV file that was used in the Pearson correlation analysis. Files in CSV format: Input and output data for the Pearson correlation analysis were all provided in the CSV file format.
Parameters for data collection	The OTUs analysed were obtained from a marine RAS for post-smolt in the first year of operation. Natural changes in operation and various RAS events occurred during the sampling period. Major RAS events were formation of skin ulcers, administration of antibiotics, farm cleaning, back flushing of the biofilter biofilm, re-inoculation of the biofilter, formation of yellow water and sharp changes in pH and salinity. The selection of fish pathogen species to be presented in the sequence database was based on the book “Bacterial fish pathogens” by Austin and Austin. Although, they used the term “Bacterial fish pathogens” in a broader sense than etiological agents, this is a well-recognized summary of fish pathogens.
Description of data collection	Samples for the amplicon library were obtained from a marine post-smolt RAS at defined sampling sites (biofilter biofilm carriers, tank wall biofilm, production water and fish skin) and sampling times (monthly). Biofilm carriers were collected by a ladle lowered into the biofilter chamber. Wall biofilm was collected by toothbrush heads passed along the wall of the production tank in study, but also, at one sampling time, from biofilter's outer chamber, the pump sump, and all production tank's depth profiles. Samples from the production water were retrieved with 0.22 μm Sterivex filters. Relative abundance information from parallel samples was averaged before the amplicon raw data were used in the Pearson correlation analysis. Sequences for the fish pathogen database were obtained from the GenBank database or from the GenBank-based database Silva 128. A total of 295 different species were included and their 6750 corresponding sequences were compiled into a fasta file and named the “Fish Pathogen Database”.
Data source location	Institution: University of Bergen City: Bergen Country: Norway Sample location: Erko settefisk AS, Stord, Norway (59°46′20.61"N 5°23′0.98"E)
Data accessibility	Repository name: Mendeley data **Fish pathogen database** Data identification number: doi: 10.17632/4zy425nty3.2 Direct URL to data: https://data.mendeley.com/datasets/4zy425nty3/2 **Pearson correlation analysis** Data identification number: doi: 10.17632/hx5dvj46rp.1 Direct URL to data: https://data.mendeley.com/datasets/hx5dvj46rp/1
Related research article	K. Drønen, I. Roalkvam, H. Nilsen, A.B. Olsen, H. Dahle, H. Wergeland, Presence and habitats of bacterial fish pathogen relatives in a marine salmon post-smolt RAS, Aquac. Reports. 26 (2022) 101312. https://doi.org/10.1016/j.aqrep.2022.101312.

## Value of the Data

• The data is of value to both the aquaculture industry, governmental fish health authorities and science.
• Open access and how to define fish pathogen relatives in a 16S rRNA gene sequence library.
• Statistical data support for exploratory microbial studies.

## 1 Objective

This data article adds value to a published research article by providing open access to data files that formed the basis of the results section of the research article. The article provides methodical information upon Pearson correlation analysis of operational taxonomic units (OTUs) from a microbiome dataset (data deposited Mendeley data) and how data was extracted from this file to present correlation data between the defined fish pathogen relatives in a separate table.

## 2 Data Description

A total of 6750 16S rRNA sequences (SSU rRNA) associated with bacterial fish pathogens were collected from the GenBank database via the Silva platform and deposited in Mendeley data with the link to data as provided in present data article. The criterion for species inclusion in the “Fish Pathogen database” was to be described in the book “Bacterial Fish Pathogens: Diseases of Farmed and Wild Fish” by Austin and Austin Austin [3]. A 16S rRNA sequence library from a post-smolt RAS (1524 sequences, 250 bp long) was previously deposited in the Mendeley database and published by Data in Brief article [2]. Sequence similarities between 90-100% were considered hits between these databases and identified the “fish pathogen relatives” as reported in the co-submitted research article [1]. Pearson correlation analysis data for all the defined OTUs (535) in the 16S rRNA sequence library were also deposited to Mendeley data with link to present paper. The OTU to relative abundance information used for the statistical analysis was given in output files from the bioinformatic pipeline that proceeded the sequencing step. The large Pearson correlation dataset was used to present information upon the defined fish pathogen relatives (Table 1 this paper), information discussed in the original research article.

**Table 1:** Pearson correlation analysis of fish pathogen-relatives identified in a marine post-smolt resirculating aquaculture system the first year of operation.

## 3 Experimental Design, Materials and Method

The original research paper describes how we went about including as many species of fish pathogens as possible in the database of full-length 16S rRNA sequences [1,3]. A total of 295 species were included in the database. These were represented by as many strain and operon variants of the 16S gene as possible, giving a total of 6750 sequences. The fasta file with the sequences was deposited in the Mendeley data repository together with a pdf file containing an overview table with all bacterial strains and their GenBank accession numbers included [4]. The link to the repository page is provided in this data article. The original research paper also described how the sequences of this fish pathogen database were aligned with the sequences in the 16S rRNA sequence library from the post-smolt RAS, and how fish pathogen relatives were defined from this.

Pearson correlations in OTU relative abundance data were analyzed for all OTUs defined in the 16S rRNA library from the RAS plant. Fewer OTUs (535) were reported in the relative abundance information table than in the sequence file (1524), this after removing OTUs that were single occurrences below 1% relative abundance. The file (CSV) with relative abundance information for sampling times and sampling sites was previously deposited to the Mendeley database with a published link in a Data in Brief article [2]. Before using this file as an input file in the correlation analysis, parallel samples were replaced with mean data and OTUs with 0% relative abundance were removed from the data set. The textual OTU information was also replaced with a numerical code. Analysis was performed on the Python 3.9.5 and Pandas 1.4.2. platforms.

Pearson's correlation coefficient is a common measure of linear association that ranges from -1 to +1, where 0 indicates no relationship. A value of +1 indicated perfect relationship, which was generally interpreted as “the more x, the more y, and vice versa.” A value of -1 represented a perfect negative relationship, with a common interpretation as “the more x, the less y, and vice versa.

Two output files (CSV raw data) were deposited Mendeley data for which the link to the repository page was provided in this data article. They differed according to the organization, either chronically by OUT or by the strength of the correlation [5]. A third pdf file (excel table) with OTU number codes for the textual information [3] was also available on the same repository page as the CSV files.

From this data set, we also obtained Pearson correlation values between OTUs in the microbiome data set that were defined as fish pathogen relatives, as presented in Table 1, data interpreted in the original research article.

## Ethics Statement

We state no ethical concerns.

## CRediT Author Statement

**K. Drønen:** Did funding acquisition, Conceptualization, Investigations, Data curation, Methodology, Writing – original draft; **I. Roalkvam:** Did data curation, Writing – review & editing; **K. Tungland:** Did formal analyzis, Software handling; **H. Dahle, H. Nilsen** and **A.B. Olsen:** Did Writing – review & editing; **H. Wergeland:** Did funding acquisition, Project administration, writing – review & editing.

## Declaration of Competing Interest

The authors declare that they have no known competing financial interests or personal relationships which have or could be perceived to have influenced the work reported in this article.

## Data Availability

Pearson correlation data (Original data) (Mendeley Data). Fish pathogen 16S rRNA database (Reference data) (Mendeley Data).
